# Respiratory motion model based correction for improving the targeting accuracy of MRI-guided intracardiac electrophysiology procedures

**DOI:** 10.1186/1532-429X-17-S1-O24

**Published:** 2015-02-03

**Authors:** Robert Xu, Prashant Athavale, Philippa Krahn, Kevan Anderson, Jennifer Barry, Labonny Biswas, Venkat Ramanan, Nicolas Yak, Mihaela Pop, Graham A Wright

**Affiliations:** 1 Physical Sciences Platform, Sunnybrook Research Institute, Toronto, ON, Canada; 2 Medical Biophysics, University of Toronto, Toronto, ON, Canada; 3 Mathematics, University of Toronto, Toronto, ON, Canada

## Background

Recently, there is an increased interest in using MRI to guide electrophysiology (EP) procedures as an alternative to X-ray fluoroscopy guidance, due to its excellent soft tissue contrast and lack of radiation. However, there exist tradeoffs between different MRI guidance schemes. Realtime 2D MR sequences are able to capture heart motion during an interventional setting, while sacrificing imaging quality, whereas high-resolution prior 3D roadmaps are static and do not reflect the respiratory motion of the heart. In this work, we explore the feasibility of deriving a motion model from these two complementary datasets, and evaluate its potential for improving the targeting accuracy of MRI-guided EP procedures.

## Methods

**Figure 1:**
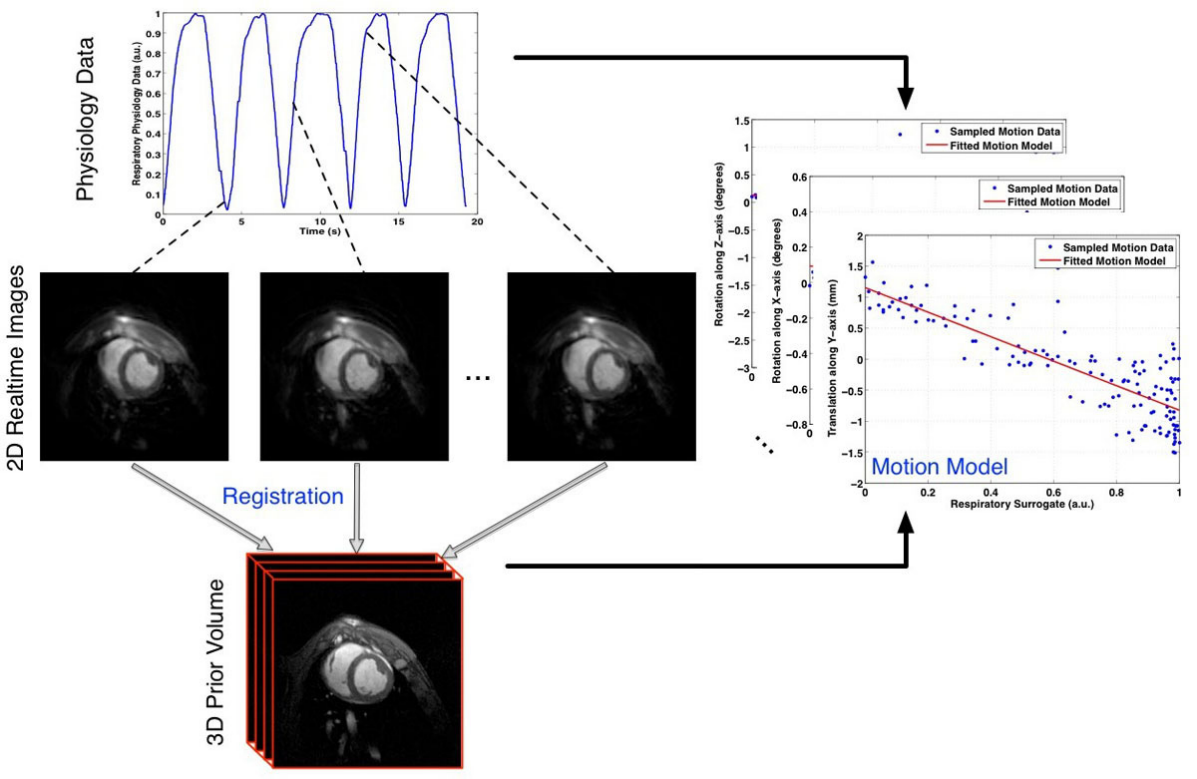
Schematic diagram of the motion model. Realtime 2D free breathing images of each pig are registered to a corresponding end expiration 3D prior volume. All images are cardiac gated and acquired along with synchronized respiratory physiology data. Image registration was used to extract motion parameters consisting of rotations and translations along the x,y,z imaging axes. Each parameter was then fitted as a linear function of the physiology data to produce a subject-specific respiratory motion model.

Motion imaging studies were performed for 4 healthy pigs, and MRI-guided catheter ablations were successfully applied in 2 pigs. Initially, a multi-slice 3D roadmap volume was acquired using a GE FIESTA imaging sequence, while the animal was mechanically ventilated and breath-held at end expiration. During the same experiment, a fast 2D balanced-SSFP spiral sequence (HeartVista) was also used to acquire free-breathing images of the heart, along with synchronized physiology data representing cardiac and respiratory phase. Under 3D prior roadmap guidance, an MR compatible and actively tracked catheter (Imricor Medical Systems) was then placed into the left ventricle (LV), while the distal tip of the catheter was continuously tracked in realtime [1], along with the corresponding physiology data. Subsequently, the catheter was maneuvered to a location along the LV wall, where an RF ablation was performed. The anatomical location of the lesion was confirmed in a post-ablation contrast enhanced IR-SSFP image. We retrospectively computed the distance between the tracked catheter ablation positions and the observed lesion center. A respiratory motion model based on multiscale registration of the ECG gated 3D prior image to 2D realtime free-breathing images was also generated [2]. The individual motion parameters were extracted and fitted as a function of the respiratory physiology data (Fig. 1). The specific motion model was then used to correct the erroneous catheter tracked positions relative to the heart wall based on their physiology data, and the distances between the motion corrected positions and the lesions were computed.

## Results

**Figure 2:**
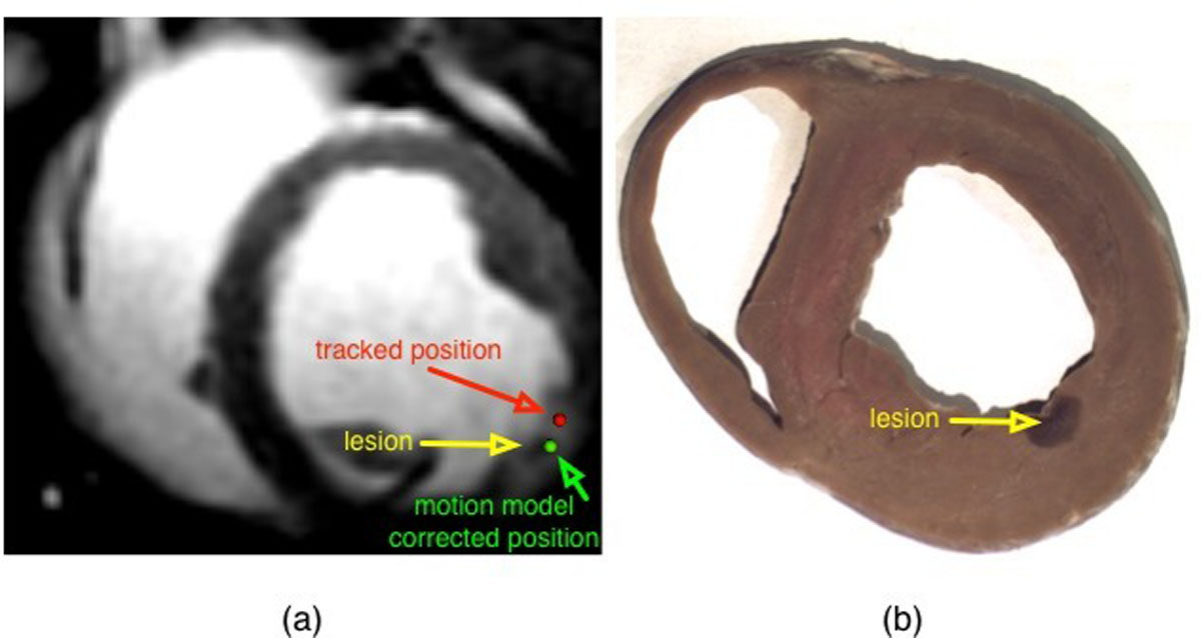
Ablation position correction. (a) Contrast enhanced IR-SSFP image showing the created lesion. Red arrow points to the location of an erroneous realtime tracked catheter tip position during RF ablation. Yellow arrow points to the actual anatomical location of the created lesion. A green arrow points to the corrected catheter position after motion model was applied to the erroneously tracked position. (b) The same lesion is shown in gross pathology at the approximate short axis slice location, after the animal was sacrificed.

The mean distance between the uncorrected catheter tracked positions and the lesion locations was 3.80±1.19 mm. After the motion correction was applied from the derived model, the mean distance between the corrected catheter positions and the lesions was 2.24±1.08 mm. An example of motion correction is shown in Fig. 2.

## Conclusions

We successfully demonstrated the feasibility to produce a data-driven model to retrospectively correct for the respiratory motion of the heart. Future work will focus on exploring the potential of the model to prospectively correct for motion and improve the ablation accuracy during MRI-guided EP procedures.

## Funding

GE Healthcare and Federal Development Agency of Canada.
